# Predictors of ‘Out-of-Pocket Expenditure’ on Routine Immunization of Under-Five Children: A Regression Analysis

**DOI:** 10.7759/cureus.11859

**Published:** 2020-12-02

**Authors:** Medha Mathur, Navgeet Mathur, Nazmeen Khan, Dewesh Kumar, Anjana Verma

**Affiliations:** 1 Department of Community Medicine, Geetanjali Medical College and Hospital, Udaipur, IND; 2 Department of General Medicine, Geetanjali Medical College and Hospital, Udaipur, Udaipur, IND; 3 Department of Community Medicine, Rajendra Institute of Medical Sciences, Ranchi, IND

**Keywords:** vaccination, vaccine hesitancy, cost, immunization schedule, health expenditure, regression analysis, public health

## Abstract

Introduction: ‘Out-of-pocket expenditure’ (OOPE) is the expenditures at the point of receiving health care directly by households which affects the economic stability of the household. When the expenditure on immunization of under-five children results in OOPE, it affects the motivation of parents for vaccination inversely.

Aim: This study was planned to evaluate the out-of-pocket expenditure and to assess predictors of OOPE on routine immunization practices.

Settings and design: This was a cross-sectional community-based study conducted from May to August 2019 at one of the immunization sites at primary health centers under a tertiary care institute of southern Rajasthan.

Material and methods: At the selected health facility, randomly 75% of beneficiaries of routine immunization were enrolled for the study. Data were collected using a pre-designed, pre-tested semi-structured questionnaire about direct and indirect expenses during the process of immunization incurred by the parents of the vaccinee by interview technique.

Results: In the study, 72.36% were infants, and 56.10% had first birth order. Mother accompanied nearly half of the vaccinee (49.6%) to the vaccination centre and the most used mode of transportation was the personal vehicle (63%). Mean loss of wages among parents/caretakers was 0.58 (±0.66) USD (median = 0.69; interquartile range (IQR) = 0.69; 95% confidence interval (CI) = 0.42-0.73) while average cost of transportation worn was 0.16 (±0.20) USD (median = 0.07; IQR = 0.21; 95% CI = 0.12-0.19) and mean duration of travelling to reach health facility was 11.87 (±7.53) minutes (median = 10; IQR = 10; 95% CI = 10.53-13.20). Vaccination at the centre was free of cost (zero USD) and no cost was incurred for drugs or registration. Average OPPE was 0.74 (±0.97) USD (median = 0.35; IQR = 0.62; 95% CI = 0.56-0.91) and regression analysis predicted significant contributors as age of vaccinee (p = 0.00; OR = 0.11; 95% CI = 0.04-0.34), religion (p = 0.04; OR = 0.34; 95% CI = 0.12-0.97) area of residence (p = 0.00; OR = 6.77; 95% CI = 2.37-19.32), birth order of vaccinee (p = 0.02; OR = 0.3; 95% CI = 0.11-0.85), longer waiting time (p = 0.03; OR = 0.16; 95% CI = 0.03-0.85), travelling time (p = 0.00; OR = 3.47; 95% CI = 1.49-8.09) and long distances (p = 0.00; OR = 10.40; 95% CI = 2.56-42.03) travelled to reach vaccination centre.

Conclusion: The hidden cost of vaccination in the form of loss of wages and time, travel cost due to stretched distance traveled by family members to accompany vaccinee to immunization facility is experienced as OOPE by the families and could be one of the impediments in vaccination coverage. Amendments in the existing policies are required to reduce this invisible cost of vaccination.

## Introduction

The term ‘out-of-pocket expenditure’ (OOPE) is defined as the expenses incurred at the point of receiving health care directly made by households [1] and is also described as health spending on hospitalization net of reimbursement [2]. In India, total health expenditure (THE) has been estimated to be 3.89% of gross domestic product (GDP) and OOPE in health care services has been estimated to be 62.6% of THE and 2.4% of GDP [1]. Social security schemes in India covers health expenditures in a limited fashion [3]. Recently launched social security scheme ‘Ayushman Bharat’ has evoked a hope to reduce health expenditures of individuals but despite various efforts by the government the health care services are not fully covered under government social security schemes which leads to OOPE. 

Out-of-pocket expenditure in health care includes traveling costs, loss of wages, cost of registration, consultation, admission, prescribed medication, food, and lodging of caregivers [4]. Such out-of-pocket expenditures may lead to catastrophic health expenditures (CHE) which are defined as household health spending exceeding 10% of household consumption expenditure or distress health financing, which is defined as a situation when a household has to borrow money or sell their property/assets or when they get contributions from friends/relatives to meet their health care expenses [2].

OOPE is not only limited to unanticipated diseases or health-related events in the household which jolts hue and cry but also escorts preventive services like immunization of under-five children in our country. 

The fully immunized child is the one who has completed primary vaccination up to one year of age while the term completely immunized is for the child who is vaccinated appropriately for his age and is aged more than two years of age. Immunization of under-five children has been done at all public health care facilities free of cost but the hidden expenditures related to immunization affect the compliance and acts as one of the undiscussed but significant reasons for vaccine hesitancy. OOPE for vaccination may include traveling costs to the health facility, loss of wages, cost of registration, and medication prescribed for adverse effects following immunization. These OOPE results in distressed financing of households and may act as one of the constraining factors for immunization [2]. The studies have indicated that although the OOPE is very low usually but it still plays a constricting factor on immunization coverage of children [5] and this results in the wide gap among actually vaccinated and reported vaccinee [6]. When there is a choice to be made between medical health care and other essential amenities in life, health takes a back seat in a developing country like India, so even low OOPE may lead to declined immunization preference [7]. The financial coverage of families for maternal and child care is already given by the center in the form of Janani Suraksha Yojna (JSY) and Janani Shishu Suraksha Karyakaram (JSSK), which indicates a step towards low OOPE in Maternal and Child Health (MCH) care but higher expenditure on other ailments like adverse events following immunization especially in rural and tribal areas has been noticed. Decentralized planning in health financing patterns may be the answer to unannounced financial burden due to expenditure on health [8-12]. Few studies are available regarding OOPE on vaccination but none in this part of the country [4,5]. Therefore, this study was designed to predict the impact of independent variables using regression analysis on the dependent variable, i.e., 'out-of-pocket expenditure' on vaccination of under-five children visiting public health care facility with their parents or caregivers in this region of the country to highlight needed changes in policy to improve the vaccination coverage.

## Materials and methods

A community-based descriptive study with the cross-sectional design was conducted from May to August 2019 at Savina, one of the primary health centers under the field practice area of the Department of Community Medicine at Geetanjali Medical College and Hospital, Udaipur, Rajasthan. Under-five children accompanied by their parents or caregivers visiting selected public health care facilities for vaccination were selected for the study and respondents were the accompanying persons with the vaccinee. Inclusion criteria were that under-five children, came only for vaccination, and parents/caretaker gave consent for the study. While children who were brought to the hospital or admitted to the health care (IPD) facility for any other ailment and non-consenting parents or caregivers were excluded from the study.

Sample size

Purposively selected PHC, Savina under the field practice area of the department of community medicine caters to 24,774 population. Records suggested that at the selected PHC on average 20 children were being vaccinated every week. We randomly selected 75% of total beneficiaries, i.e., 15 beneficiaries attending vaccination facilities per week were interviewed for eight weeks of duration. So, based on the above calculation 120 beneficiaries were be included in the study [13]. Considering a 10% non-response rate, a total of 132 study subjects were enrolled for the study.

Study tool

The data was collected using a pre-designed pre-tested semi-structured questionnaire which had multiple sections to help the researcher achieving the aims and objectives of the study. The tool had sections namely socio-demographic profile of the parent-child, individual vaccine information, the direct cost of vaccination in the form of the cost incurred in registration, vaccine cost, other consumables cost, and cost of medication incurred in case of any adverse event following immunization (AEFI) and indirect cost like the self-reported cost of traveling, loss of wages while attending the immunization center, waiting time and any other cost incurred in the form of stay and food. Travel cost was calculated by self-reported cost of transportation of the accompanying persons based on the mode of transportation. The loss of income for adults was calculated by multiplying the self-reported duration spent (traveling duration+ waiting duration + time spent for vaccination) with daily wages.

The amount calculated in the form of loss of wages and traveling cost were actually taken in INR but converted to USD for making uniformity while comparing with available data [4]. The exchange rate considered for the base year 2019 was 1 USD= 72.12 INR [14]. 

Strategy

Indirect cost or cost-related to income loss was estimated based on the lost productivity (in hours) of those who (parents or caretakers) accompanied children for vaccination. The hours not worked were counted as hourly income loss. The loss of income for adults was calculated by multiplying the self-reported time and daily wage rates.

One to one interview technique was used by an investigator for data collection and questions were asked after the process of vaccination is over, and to avoid an increase in the duration of the vaccination process, interview time was deducted from the total duration of the vaccination process. The questionnaire was translated into ‘Hindi’ and validated. After data collection, responses were again translated into English, and data was entered in MS office Excel. Collected data were analyzed using Statistical Package for Social Sciences (SPSS) Statistics for Windows, version 24 (IBM Corp., Armonk, N.Y., USA), and relevant tests of significance were applied. Regression analysis was done to establish the relation between OOPE and contributing variables.

Ethical considerations

The study was started only after obtaining ethical clearance from the Institutional Ethics committee (IEC) of the institute (GU/HREC/EC/2019 dated 01.02.2019).

## Results

A total of 132 participants were enrolled in the study, but nine participants were dropped from analysis due to incomplete data. So, a total of 123 study subjects were assessed for their sociodemographic characteristics, child’s immunization details, and direct and indirect cost related to vaccination.

Under-five children in the study were distributed from zero to 60 months of age. The mean age of the children included in the study was 10.45 (±4.34) months (median eight months and range 1.5 to 55 months). Children from zero to 11 months were 89 (72.36%), from 12 to 24 months were 19 (15.45%) and from 24 to 60 months were 15 (12.19%).

Among 123 under-five study subjects, i.e., vaccinee who attended vaccination center, 65 (52.85%) were male while 58 (47.15%) were females. As per distribution of religion, study subjects who followed Hinduism were 94 (76.43%), Muslims were 11 (8.94%) while others were 18 (14.63%) where others included Sikh, Christians, Buddhists, and Jains. Most of the participants who visited the vaccination facility belonged to rural areas, i.e., 90 (73.17%) while urban residents were only 33 (26.83%). 

Under-five vaccinee belonged to both joint 67 (54.47%) and nuclear families 56 (45.53%). As per the modified Kuppuswamy’s socioeconomic classification, most of the respondents were in ‘upper- and lower-middle’ classes (39.02%, 36.59%, respectively) while 14.64% lied in ‘lower and upper-lower’ class and only 9.76% were in ‘upper’ class as shown in Table 1. Of the total, 69 (56.10%) vaccinee had first birth order while 42 (34.14%) and 12 (9.76%) had birth order second and third or higher respectively (Table 1).

**Table 1:** Sociodemographic details of the study subjects

S.N.	Characteristic		Frequency (%)
1	Age (months)	0-11	89 (72.36)
		12-24	19 (15.45)
		24-60	15 (12.19)
2	Gender	Male	65 (52.85)
		Female	58 (47.15)
3	Caste	General	35 (28.5)
		OBC	34 (27.6)
		SC	35 (28.5)
		ST	19 (15.4)
4	Religion	Hindu	94 (76.43)
		Muslim	11 (8.94)
		Others	18 (14.63)
5	Area of residence	Rural	90 (73.17)
		Urban	33 (26.83)
6	Type of family	Nuclear family	56 (45.53)
		Joint Family	67 (54.47)
7	Socioeconomic status	Class I	12 (9.76)
		Class II	48 (39.02)
		Class III	45 (36.58)
		Class IV	9 (7.32)
		Class V	9 (7.32)
8	Birth order of the vaccinee	1	69 (56.10)
		2	42 (34.14)
		3 or more	12 (9.76)
9	Number of accompanying persons with the vaccinee	1	61 (49.60)
		2	49 (39.84)
		3 or more	13 (10.56)

The vaccinee accompanied by parents or relatives were assessed in the study, and it was found that 61 (49.60%) subjects had single company, while 49 (39.84%) had two accompanying people and three or more accompanying family members were observed with only 13 (10.56%) subjects. In 96 cases, the mother accompanied the child, while 24 children were brought to the health facility by the father, and only three were accompanied by other relatives. It was found that 50 (40.65%) vaccinee was accompanied by one of the earning members of the family to the health facility for routine immunization.

The vaccination status of the child is highly influenced by the educational status of the mother. In this study, the educational status of the mother of vaccinee was assessed and found that nine (7.32%) mothers were illiterate, while 78 (63.42%) mothers had only school education while 19 (15.44%) were graduate and 17 (13.82%) were postgraduate. On analyzing the occupation of father of vaccinee it was found that 45 (36.58%) were employed in service, 42 (34.15%) were businessmen, 33 (26.83%) were laborers and only three (2.44%) were involved in farming.

Various modes of transportations were utilized by respondents for accessing the vaccination center. Most commonly, people utilized private vehicles (63%) followed by walking (27%) and public transportation (10%). Amongst private vehicles, two-wheeler motorbikes were most frequently (97%) used and only a few (3%) used four-wheeler vehicles for transportation. 

The direct and indirect variables which account for the losses due to the process of immunization of a child of household are in the form of time spent, distance traveled, and loss of wages of earning members. Average distance travelled by the parents for immunization was 2.2 (±2.82) km ranging from 0.5 to 15 km to the health facility (median = 1; interquartile range (IQR) = 1.50; 95% confidence interval (CI) = 1.70-2.69). Mean loss of wages among those who accompanied their child for vaccination was 0.58 (±0.66) USD ranging from zero to 2.77 USD (median = 0.28; IQR = 0.69; 95% CI = 0.42-0.73). The average cost of transportation worn for immunization of the vaccinee was 0.16 (±0.20) USD ranging from zero to 1.11 USD (median = 0.07; IQR = 0.21; 95% CI = 0.12-0.19). The average duration of traveling to reach the health facility was 11.8 (±7.53) minutes with a range from five to 40 minutes (median = 10; IQR = 10; 95% CI = 10.53-13.20). Average waiting time for vaccination at the health facility was 18.1 (±14.57) minutes, where few respondents reported waiting time of five minutes while a few reported waiting for durations up to two hours (median = 15; IQR = 10; 95% CI = 15.58-20.76). The average total duration spent at the health facility for vaccination (waiting duration + duration of vaccination+ observation time) was nearly 28.8 (±16.6)minutes. The mean total duration spent on the vaccination i.e. time spent at the health facility and traveling duration was found to be 40.6 minutes (Table 2).

**Table 2:** Variables responsible for direct and indirect losses due to the immunization process. SD: standard deviation; IQR: interquartile range; CI: confidence interval; USD: United States dollar

Variables	Productivity loss					
	Mean (±SD)	Median	Range	IQR	95% CI for Mean	
					Lower	Upper
Travel time (minutes)	11.87 (±7.53)	10	5-40	10	10.53	13.20
Waiting time (minutes)	18.17 (±14.57)	15	5-120	10	15.58	20.76
Distance traveled (kilometers)	2.20 (±2.82)	1	0.5-15	1.50	1.70	2.69
Traveling cost (USD)	0.16 (±0.20)	0.07	0-1.11	0.21	0.12	0.19
Loss of wages	0.58 (±0.66)	0.28	0-2.77	0.69	0.42	0.73
Out-of-pocket expenditure (USD)	0.74 (±0.97)	0.35	0-3.47	0.62	0.56	0.91

The cost of vaccination at the public health facility was 0 (zero) USD which included the cost of registration, cost of vaccines, cost of administration of vaccines, and cost of any drugs prescribed after vaccination. No expenditure on vaccination by the family member implies successful implementation of the national immunization program which provides costless vaccines at all levels. Still, the ‘out-of-pocket expenditure’ on vaccination reported being approximately 0.74 USD which accounted for the cost incurred for traveling and loss of wages while accompanying the ward for vaccination (Table 2).

Table 3 illustrates the logistic regression analysis of the factors associated with OOPE. It includes p-values, odds ratios (ORs), and 95% confidence intervals for each feature using binary logistic regression.Gender of the vaccine (p = 0.145), caste (p = 0.743), family type (p = 0.216), socioeconomic status (p = 0.069) and accompanying persons (p = 0.094) with the vaccinee to the health care facility did not show a significant association with OOPE. Whereas Infantile age group (p < 0.00; OR = 0.11; 95% CI = 0.04-0.34), religion (p = 0.044; OR = 0.34; 95% CI = 0.12-0.97), first birth order of vaccinee (p = 0.024; OR = 0.30; 95% CI = 0.10-0.85)showed lower OOPE while residents of urban area (p < 0.00; OR = 6.77; 95% CI = 2.37-19.32) showed higher OOPE during the process of vaccination. Waiting time of less than 15 minutes (p = 0.032; OR = 0.15; 95% CI = 0.03-0.85) showed lesser out-of-pocket expenditure while travelling time of more than 15 minutes (p = 0.004; OR = 3.47; 95% CI = 1.49-8.09) showed approximately 3.5 times higher OOPE and distance travelled of more than 5 km (p = 0.001; OR = 10.40; 95% CI = 2.57- 42.03) to reach the health facility for vaccination was found to have a significantly higher association with the out-of-pocket expenditure on the routine immunization.

**Table 3:** Logistic regression analysis of variables with out-of-pocket expenditure on vaccination. ^a^Reference category. ^b^Includes Other Backward Classes, Schedule Caste and Tribes. ^c^Includes Muslims, Sikh, Buddhist, Jains, etc. ^d^Includes class I & II. ^e^Class III, IV, V. ^f^Includes three-generation families. ^g^p-Value is statistically significant. OR: odd ratio; CI: confidence interval

Variables		p-Value	OR	95% CI for OR	
				Lower	Upper
Age (months)	0-11	0.000^g^	0.11	0.04	0.34
	12-60^a^				
Gender	Male	0.145	2.16	0.76	6.13
	Female^a^				
Caste	General	0.743	1.19	0.41	3.43
	Others^b,a^				
Religion	Hindu	0.044^g^	0.34	0.12	0.97
	Others^c,a^				
Area of residence	Urban	0.000^g^	6.77	2.37	19.32
	Rural^a^				
Socioeconomic status	Upper^d^	0.069	2.62	0.92	7.44
	Lower^e,a^				
Type of family	Nuclear	0.216	0.51	0.18	1.46
	Joint^f,a^				
Birth order of vaccinee	1st	0.024^g^	0.30	0.10	0.85
	≥ 2nd^a^				
Accompanying persons	One	0.094	0.41	0.14	1.16
	≥ Two				
Waiting time (minutes)	<30	0.032^g^	0.15	0.03	0.85
	≥ 30^a^				
Travelling Time (minutes)	≥ 15	0.004^g^	3.47	1.49	8.09
	<15^a ^				
Distance travelled (kilometres)	>5	0.001^g^	10.40	2.57	42.03
	< 5^a ^				

## Discussion

The immunization program of India is the largest in the world and around 26.7 million infants and 30 million pregnant women are covered annually. Despite continuous progress, it has been noted that there is a slow rise in routine childhood vaccination coverage. An estimated 38% of children failed to receive all basic vaccines in the first year of life or in other words largest immunization program in the world covers only 62% of children in the first year of their lives. Vaccination coverage in India after all the efforts from the government including ‘Mission Indradhanush’ and ‘Intensified Mission Indradhanush’ has increased 6.9% for fully immunized children since 2016 [6,15-17].

Various researches have reported reasons for low vaccination coverage ranging from sociocultural factors to vaccine hesitancy due to ignorance and side effects. This study tried to highlight the factors contributing to the out-of-pocket expenditure incurred by the families during the process of routine vaccination of children. Factors studied were age and birth order of the vaccinee, caste, religion, area of residence, and socioeconomic status of the family as demographic variables. other factors considered in the study were the number of accompanying persons to the vaccination facility with the child, loss of wages, traveling time, waiting time and total distance traveled to reach the health facility. 

Factors contributing to lesser OOPE in the study were infantile age group, religion, and first birth order of the vaccine while direct contributors of OOPE were longer waiting and traveling time along with the farther distance of vaccination center.

The average traveling time was estimated to be 11.8 minutes in the current study as compared to a study conducted to assess OOPE in cholera vaccination where beneficiaries traveled nearly 26.5 minutes to reach the vaccination booth and their waiting time was 15.7 minutes as compared to 18 minutes in the current study [4]. The total duration spent on vaccination was found to be 40.8 minutes in the current study. Logistic regression analysis showed that more waiting time, traveling time, and distance have contributed significantly to higher OOPE in the current study. Urban residents were also found to have higher OOPE as compared to their rural counterparts.

In the context of modes of transportation, in a study conducted by Mogasale et.al. on oral cholera vaccination, it was found that 92% of household members walked from their homes to vaccination booths, and the remainder used bicycles (10%) or motorbikes (2%) and one household used a car in contrast to current study where 27% beneficent families walked, 10% used public transport and 65% traveled through private vehicles [4]. The time duration lapsed in traveling is contributing to out-of-pocket expenditure in the same way as distance traveled is a part of out-of-pocket expenditure in beneficiaries. The occupation of parents/accompanying person was affected by time spent for vaccination and loss of wages was 0.58 (±0.66) USD on an average in the study which was calculated as per the reported daily wages of the people multiplied by the duration spent on vaccination. But the factor that was not considered in the study was that few study participants took paid leave for the purpose and did not face any loss of wages directly. But daily wage workers suffered a direct loss in wages.

Another important point was the duration spent on vaccination was prolonged by the interview for this study purpose but to eliminate the influence of this, the duration of the interview was deducted while calculating the total duration spent on vaccination to maintain uniformity in the calculation.

As claimed by the National Immunization Program, free delivery, and administration of vaccines, our study found the same and participants experienced no expenditure in the actual procedure of vaccination [6]. 

So, it was found that out-of-pocket expenditure in the study was mainly because of the rural area, traveling expenses and distance traveled as time spent in the process of vaccination. Although free vaccination has improved vaccination coverage in the country, the above said out-of-pocket expenditures may act as reasons for vaccine hesitancy.

In a country like India where social security schemes are not universal, expenditure (out of pocket) over important items like under-five immunization may bring financial stress to the family and maybe an important reason to delay or deny vaccination [5]. Social and health insurance policies should cover routine immunization as it is an important preventive strategy to combat vaccine-preventable communicable diseases.

## Conclusions

The out-of-pocket expenditure is one of the hidden expenditures which is not included in the vaccination cost during immunization. To conclude the immunization of under-five children has proved to have the out-of-pocket expenditure of considerable amount, which is in the form of area of residence, traveling cost, traveling distance, and indirectly loss of wages due to time spent in traveling, waiting, and process of vaccination. Through this study, we could conclude that it could be one of the reasons for lower vaccination coverage, and vaccine hesitancy can be associated with this extra expenditure. The health providers are given allowances for vaccination services but still, policymakers should take ‘out-of-pocket expenditure’ of the families of vaccinee into consideration because it may lead to catastrophic health expenses shattering the economy of the recipient family. Our government has already started health insurance benefits for a large segment of the population, and out-of-pocket expenditure due to immunization should be included under that initiative. The traveling cost can be reduced by providing quality services in the periphery. Further researches will be needed to assess the out-of-pocket expenditure in other parts of the country as this research was conducted only in the selected area of Udaipur district and the results can only be generalized after additional shreds of evidence.
